# Sporotrichosis in the Highlands of Madagascar, 2013–2017^1^

**DOI:** 10.3201/eid2510.190700

**Published:** 2019-10

**Authors:** Tahinamandranto Rasamoelina, Danièle Maubon, Onivola Raharolahy, Harinjara Razanakoto, Njary Rakotozandrindrainy, Fetra Angelot Rakotomalala, Sébastien Bailly, Fandresena Sendrasoa, Irina Ranaivo, Malalaniaina Andrianarison, Benja Rakotonirina, Abel Andriantsimahavandy, Fahafahantsoa Rapelanoro Rabenja, Mala Rakoto Andrianarivelo, Lala Soavina Ramarozatovo, Muriel Cornet

**Affiliations:** Centre d’Infectiologie Charles Mérieux, Université d’Antananarivo, Antananarivo, Madagascar (T. Rasamoelina, F.A. Rakotomalala, M.R. Andrianarivelo);; University Grenoble Alpes, Grenoble, France (D. Maubon, S. Bailly, M. Cornet);; Unité de Soins de Formation et de Recherche, Centre Hospitalier Universitaire Joseph Raseta Befelatanana, Antananarivo (O. Raharolahy, H. Razanakoto, F. Sendrasoa, I. Ranaivo, M. Andrianarison, F.R. Rabenja, L.S. Ramarozatovo);; Unité Para-clinique de Formation et de Recherche, Centre Hospitalier Universitaire Joseph Ravoahangy, Antananarivo (N. Rakotozandrindrainya);; Université d’Antananarivo, Antananarivo (B. Rakotonirina, A. Andriantsimahavandy);; Pavillon Spécial A Centre Hospitalier Universitaire de Befelatanana, Antananarivo (L.S. Ramarozatovo)

**Keywords:** Sporotrichosis, epidemiology, prevalence, molecular diagnosis, clinical presentation, outcome, *Sporothrix schenckii*, highlands, Madagascar, fungi, zoonoses

## Abstract

Sporotrichosis is a saprozoonotic fungal infection found mostly in tropical and subtropical areas. Few case reports in Madagascar have been published. To document sporotrichosis epidemiology in Madagascar, we conducted a cross-sectional study. During March 2013–June 2017, we recruited from select hospitals in Madagascar patients with chronic cutaneous lesions suggestive of dermatomycosis. Sporotrichosis was diagnosed for 63 (42.5%) of 148 patients. All but 1 patient came from the central highlands, where the prevalence was 0.21 cases/100,000 inhabitants. Frequency was high (64.7%) among patients <18 years of age. Sporotrichosis was diagnosed for 73.8% of patients with arm lesions, 32.3% with leg lesions, and 15.4% with lesions at other sites. Molecular identification identified 53 *Sporothrix schenckii* isolates. Among the 32 patients who were followed up, response to itraconazole was complete or major for 15 and minor for 17. Overall, endemicity of sporotrichosis in Madagascar was high, concentrated in the highlands.

Sporotrichosis is a chronic fungal infection of humans and animals, found mostly in tropical and subtropical regions. The causal fungi develop in the soil or on plants and infect mammals through wounds, either directly (wounds from spiky plants or thorns) or through contact with contaminated soil or infected animals. Thus, sporotrichosis is a so-called implantation mycosis, affecting principally rural populations, particularly those who work with bare hands and feet (*1*–*3*)*.* The disease is caused by a dimorphic fungus of the genus *Sporothrix*. These fungi display a high degree of genomic diversity, leading to the description of at least 6 cryptic species: *S. schenckii*, *S. brasiliensis*, *S. globosa*, *S. luriei*, *S. mexicana*, and *S. albicans* (formerly *S. pallida*). *S. mexicana* and *S. albicans* are mostly environmental (saprophytic and nonpathogenic) (*4*–*7*)*.* The infection generally occurs as a lymphocutaneous form with an ulcerated subcutaneous nodule at the inoculation site and similar secondary lesions arising along the lymphatic route (*1*,*5*)*.* Mucosal or primary pulmonary forms are less common (*8*)*.* Some cases occur as disseminated forms with multiorgan involvement, most notably in HIV-infected persons (*9*,*10*).

Sporotrichosis is widespread throughout the world; several areas of known hyperendemicity are Brazil, Mexico, Peru, and China. Outbreaks from various environmental sources, involving thousands of persons, have been reported (*1*–*3*,*11*,*12*). In Brazil, a large zoonotic outbreak associated with cats is ongoing; it has been suggested that a strain of *S*. *brasiliensis* with enhanced virulence is involved (*13*–*15*).

In Madagascar, no epidemiologic data are available for evaluation of the sporotrichosis burden. Dermatologists and infectious disease specialists have reported encountering a large number of suspected cases during their routine medical consultations; however, the cases have not been biologically confirmed. In 2007, the dermatology department of Antananarivo University Hospital in the capital of Antananarivo confirmed a series of cases and reported 1 case (*16*). Since 2013, we conducted a cross-sectional study to document the current epidemiology of this fungal infection in Madagascar. To diagnose, confirm, and identify the fungal species responsible, we used conventional mycology and molecular biology methods, including matrix-assisted laser desorption/ionization-time of flight (MALDI-TOF) mass spectrometry. We describe the average annual prevalence and clinical presentation of sporotrichosis in Madagascar, along with patient outcomes. We also report the species-level identification, genetic relatedness, and antifungal susceptibility of the clinical isolates.

## Materials and Methods

### Study Design and Patient Recruitment

**Figure 1:**
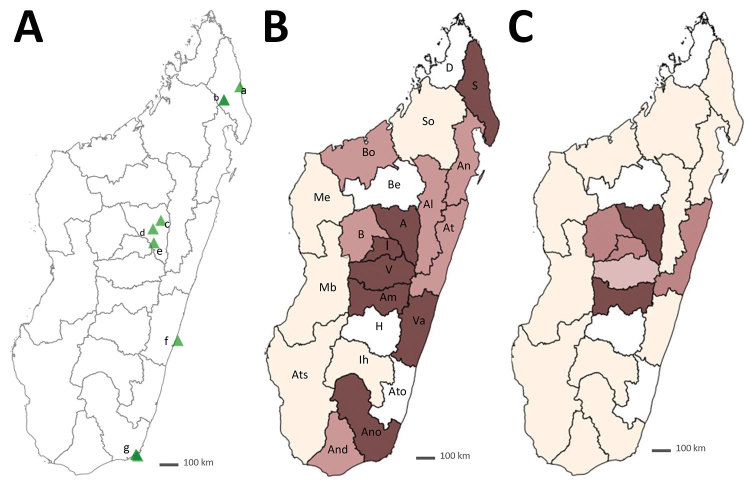
Recruitment of patients with chronic cutaneous and subcutaneous lesions and annual prevalence of sporotrichosis, Madagascar, March 2013–June 2017. A) Recruitment sites. Sava Region: a) Centre Hospitalier de Référence Régionale, Sambava District; b) Centre Hospitalier de District and Hôpital Adventiste, Andapa District; Analamanga Region: c) Centre de Santé de Base, Alakamisy-Anjozorobe, Anjozorobe District; d) Centre Hospitalier Universitaire Joseph Ravoahangy Befelatanana, Antananarivo District; e) Centre de Santé de Base, Andramasina District; Vatovavy Fitovinany Region: f) Fondation Médicale Ampasimanjeva, Manakara District; Anosy Region: g) Centre Médical Tolagnaro, Centre Hospitalier de Référence Régionale, Tolagnaro and Hôpital Luthérien Manambaro, Tolagnaro District. B) Patients’ region of origin, from north to south: D, Diana; S, Sava; I, Itasy; A, Analamanga; V, Vakinankaratra; B, Bongolava; So, Sofia; Bo, Boeny; Be, Betsiboka; Me, Melaky; Al, Alaotra-Mangoro; At, Atsinanana; An, Analanjirofo; Am, Amoron’I Mania; H, Haute Matsiatra; Va, Vatovavy-Fitovinany; Ato, Atsimo; Ih, Ihorombe; Mb, Menabe; Ats, Atsimo Andrefana; And, Androy; Ano, Anôsy. Number of patients recruited: dark brown, n>6; medium brown, n = 3–5; beige, n<3; white, missing. Black outlines indicate regional boundaries. C) Annual prevalence of sporotrichosis, showing origins of the 63 sporotrichosis patients described in this study. Prevalence per 100,000 inhabitants: dark brown, >0.2; medium brown, 0–0.2; light brown, <0.1; beige, 0; white, missing. Black outlines indicate regional boundaries.

We performed a cross-sectional study of patients with clinically suspected sporotrichosis or another chronic dermatomycosis (i.e., unique or multiple nodular, budding, wart-like, or plaque-like skin lesions following a lymphatic vessel, with or without ulceration, enduring for >1 month). Patients were recruited during March 2013–June 2017 (4 years and 3 months, hereafter referred to as a 4-year period) from the Dermatology Department of the Joseph Raseta Befelatanana University Hospital (CHUJRB) in Antananarivo or during advanced consultation campaigns in regional hospitals (Figure 1, panel A). These campaigns were preceded by announcements made via radio, social media, and posters that invited patients with cutaneous or subcutaneous lesions to come to these consultations. Healthcare providers completed clinical and demographic information forms that asked about the patient’s age, sex, and occupation; the probable area of ​​contamination; anatomic location and appearance of the lesions; and treatments received. We excluded from the study 2 patients for whom this information could not be obtained. The study was approved by the Ethics Committee for Biomedical Research of the Ministry of Public Health of Madagascar (authorization no. 66-MSANP/CE). After sampling, treatment (200 mg/d of itraconazole) was provided free of charge to all recruited patients for at least 2 months.

### Statistical Methods

We compared sporotrichosis cases and other nonsporotrichosis cases by using χ^2^ or Fisher exact tests for qualitative variables and Student *t* tests for quantitative variables and a χ^2^ test for trend to compare annual frequencies. We estimated the average annual prevalence as the mean number of cases per year (considering the period as a 4-year period) over the mean number of inhabitants. The mean number of inhabitants was calculated from the available figures in 2013 (obtained from the National Institute of Statistics of Madagascar, https://www.instat.mg) and adjusted according to the estimate of the World Bank for demographic growth of 2.7% per year (https://donnees.banquemondiale.org/indicateur/SP.POP.GROW)*.* In our evaluation of the prevalence in the highlands of Madagascar, we excluded the region of Haute Matsiatra because no patients were recruited from there. We analyzed data and generated maps by using EpiInfo version 7.2.2.1 (https://www.cdc.gov/epiinfo/index.html) and RStudio version 1.0.153 (https://www.r-project.org*)*.

### Case Definitions

We used a list of clinical, mycologic, histologic, and prognostic criteria to classify cases in this study (Table 1). Cases were identified after a monthly consultation among clinicians of the Department of Dermatology of the CHUJRB and teams of mycologists from the Charles Mérieux Infectiology Center of Antananarivo, Madagascar, and Université Grenoble Alpes, Grenoble, France.

**Table 1:** Criteria used to classify cases of sporotrichosis in the Highlands of Madagascar, 2013–2017*

Criteria	Description
Clinical	
Major	Cutaneous: lymphocutaneous form defined as a papule or pustule or a subcutaneous nodule at the inoculation site, then ulceration with erythematous edges and purulent secretion. Secondary lesions arise along the path of regional lymphatic vessels. Fixed or cutaneously disseminated.
	Extracutaneous: disseminated, osteoarticular, ocular.
Minor	Mucosal: nasal septum, with bloody secretions and detachment of crusts. Conjunctivitis, with granulomatous lesions accompanied by a serous-purulent discharge, redness, lid edema, and preauricular and submandibular lymph node enlargement.
	Primary pulmonary sporotrichosis: similar to that of tuberculosis. Radiologic patterns include cavitary disease, tracheobronchial lymph node enlargement, and nodular lesions. Vegetative, verrucous, infiltrated plaque, or tuberous lesion.
Mycologic and histologic	
Major	Molecular evidence of *Sporothrix schenckii* on PCR with specific primers (targeting topoisomerase II) or ITS sequencing, directly from clinical samples or from a positive culture of a fungus morphologically suggestive of *Sporothrix* spp.
	MALDI-TOF mass spectrometry identification of *S. schenckii* from a positive culture of a fungus morphologically suggestive of *Sporothrix* spp.
Minor	Budding yeast cells with the characteristic cigar-shaped buds observed on direct microscopic examination or histologic analysis.
	Direct examination of pus and/or histologic analysis showing asteroid bodies (Splendore-Hoeppli reaction).
	Positive culture of a fungus morphologically suggestive of *Sporothrix* spp. from a clinical sample without molecular or MALDI-TOF mass spectrometry confirmation.
Classification	
Confirmed	>1 of the major clinical criteria and >1 of the major mycologic criteria or 1 minor clinical criterion and >1 of the major mycologic criteria.
Probable	>1 of the major clinical criteria and 1 minor mycologic or histologic criterion and a complete or partial response to antifungal therapy.
Possible	>1 of the major clinical criteria without any (major or minor) mycologic or histologic criteria or >1 of the minor clinical criteria without any (major or minor) mycologic or histologic criteria and a complete or partial response to antifungal therapy.
Clinical response to antifungal therapy	
Cure	Complete resolution of all lesions.
Major response	Substantial improvement of most lesions with a substantial decrease in subcutaneous nodules.
Minor response	Mild improvement of most lesions with a smaller decrease in subcutaneous nodules than for a major response.
Failure	Stabilization of the lesions after >3 months of antifungal therapy or worsening of the lesions after >3 months of antifungal therapy.

*ITS, internal transcribed spacer; MALDI-TOF, matrix-assisted laser desorption/ionization time-of-flight.

### Clinical Samples

After obtaining patient consent, we collected specimens consisting of biopsy material, pus, or flakes of skin from all patients. All samples were sent to the laboratory of the Charles Mérieux Infectiology Center of Antananarivo, where they were processed either immediately or after 24 to 48 hours of storage at 2°–8°C.

### Mycologic Analyses

We directly examined the clinical specimens under a microscope (*5*,*11*). The samples were used to inoculate Sabouraud medium supplemented with chloramphenicol and incubated at 30°C for 2–3 weeks. For positive cultures, we identified the fungal isolates morphologically, extracted DNA, and froze the culture at −80°C.

### Molecular Analyses

We used the QIAamp DNA Blood Mini Kit (https://www.qiagen.com) according to the manufacturer’s instructions for DNA purification. Colonies and biopsies were crushed before processing. PCR amplification was performed in 2 steps. The first step comprised 2 panfungal PCRs targeting internal transcribed spacer (ITS) regions with the primers ITS1/ITS4 and D1D2 with the primers NL-1/NL-4 and NL-3/NL-4 (*17*–*19*). The second step was a specific *S. schenckii* PCR targeting the topoisomerase II gene with SSHF31/SSHR97 primers (*20*)*.* We sequenced panfungal PCR products by LGC Genomics GmbH (https://www.biosearchtech.com) by using the same primers and aligned the sequences obtained with the reference sequences in the International Society of Human and Animal Mycology (ISHAM) Barcoding Database (http://its.mycologylab.org) (*17*) for the ITS region and the National Center for Biotechnology Information database for the D1D2 and ITS regions. We constructed the phylogenetic tree by using MEGA7 software (https://www.megasoftware.net).

### MALDI-TOF Mass Spectrometry Analysis

We created a main spectrum profile (MSP) in-house *Sporothrix* library on the Microflex mass spectrometer, according to the MALDI Biotyper version 1.1 MSP creation protocol (Bruker Daltonicks, https://www.bruker.com) from a reference strain of *S. schenckii* (IHEM 3787) and 18 isolates formally identified by DNA sequencing or the specific *S. schenckii* topoisomerase II PCR. We cultured isolates under 3 conditions: in Sabouraud–chloramphenicol agar for 4–7 days at 30°C, in liquid Sabouraud medium for 2–4 days at 25–30°C with shaking, and on solid peptone dextrose agar (YPD; Sigma Aldrich, https://www.sigmaaldrich.com) for 4–5 days at 30°C. This library was validated with the IHEM 3774 reference strain and 35 clinical strains obtained during this study but not used to create the MSPs. We used the ethanol formic acid extraction procedure on YPD subcultured strains in accordance with the MALDI BiotyperIVD protocol version 1.6. We compared the spectra obtained with the Bruker Taxonomy (7,815 entries), Bruker Filamentous Fungi (364 MSP), NIH mold (365 profiles) (*21*), and our new MSP in-house *Sporothrix* library, generating identification scores with the following quality criteria: score >2, species-level identification; score <1.7 to <2, genus-level identification; score <1.7, no identification. In addition, we applied an external control by submitting both our MSP in-house *Sporothrix* and identification spectra to an independent online database, MSI (https://msi.happy-dev.fr)*.* This database contains reference spectra for *S.* *schenckii*, *S. brasiliensis*, *S. urviconia*, *S. fungorum*, *S. globosa*, *S. humicola*, *S. inflata*, *S. insectorum*, *S. pallida*, *S. stenoceras*, and *S. variecibatus* (*22*)*.*

### Susceptibility to Antifungal Drugs

To determine the MICs of antifungal agents, we used the Clinical and Laboratory Standards Institute (https://clsi.org) protocol for filamentous fungi on mycelial strains after subculture at 30°C (*23*). We tested the following agents at the concentrations indicated: posaconazole and isavuconazole (0.016 to 8 μg/mL), amphotericin B and itraconazole (0.006 to 32 μg/mL), and terbinafine (0.008 to 4 μg/mL). We determined MICs after 72 hours of culture at 30°C with a 100% inhibition endpoint for all drugs except terbinafine, for which the endpoint was 80%, as described by Espinel-Ingroff et al. (*24*)*.*

## Results

### Demographic and Clinical Characteristics of the Patients

#### Total Cohort

During March 2013–June 2017, we recruited 148 patients with chronic cutaneous or subcutaneous lesions. Median patient age was 39 years (interquartile range 22–53 years). Male patients predominated (n = 111, 75%), and the largest number of patients (n = 118, 79.7%) was enrolled by CHUJRB, the permanent recruitment center (Figure 1, panel A, triangle d). An analysis of the geographic origin of recruited patients showed that most (n = 90, 60.8%) came from the highlands, followed by the regions of the northeast (n = 23, 15.5%), east and southeast (n = 16, 10.8%), south and southwest (n = 13, 8.8%), and west (n = 6, 4.1%) (Figure 1, panel B). The largest proportion of recruited patients worked in agriculture (n = 76, 51.3%), followed by the service sector (n = 31, 21%); other patients were students (n = 20, 13.5%), craftsmen (n = 14, 9.5%), or unemployed (n = 7, 4.7%). Lesions were located mostly on the legs (62.8%) and arms (28.3%).

#### Patients with Sporotrichosis

At the first consultation, 47 of the 148 patients had clinically suspected sporotrichosis. A diagnosis of sporotrichosis was recognized for 63 (42.5%) patients, confirmed for 53 (35.8%), and possible for 10 (6.7%). The frequency of sporotrichosis remained stable from 2013 through 2017 (37.5%–64%, p = 0.16; Table 2). From 2014 through 2016, the mean (+SD) number of annual sporotrichosis cases was 14 (+5.1).

**Table 2:** Description of sporotrichosis cases in a cohort of patients with chronic cutaneous and subcutaneous lesions, Madagascar, March 2013–June 2017*

Characteristic	Sporotrichosis, no. (%), n = 63	Other, no. (%), n = 85	p value
Recruitment period			0.16†
2013, from March 1	6 (9.6)	10 (11.8)	
2014	16 (25.4)	12 (14.1)	
2015	21 (33.3)	26 (30.6)	
2016	13 (20.6)	23 (27.1)	
2017, until May 31	7 (11.1)	14 (16.4)	
Age range, y			0.2‡
3–18	11 (17.5)	6 (7.1)	
19–33	15 (23.8)	16 (18.8)	
34–48	17 (27.0)	30 (35.3)	
49–63	12 (19.1)	17 (20.0)	
64–80	8 (12.7)	16 (18.8)	
Sex			0.29‡
M	44 (69.8)	67 (78.8)	
F	19 (30.2)	18 (21.2)	
Lesion location			<0.0001‡
Leg	30 (47.6)	63 (74.2)	
Arm	31 (49.2)	11 (12.9)	
Other§	2 (3.2)	11 (12.9)	
Occupation			0.07‡
Farmer	33 (52.4)	43 (50.6)	
Service sector	8 (12.7)	23 (27.1)	
Student	9 (14.3)	11 (12.9)	
Craftsman/tradesman	10 (15.8)	4 (4.7)	
Unemployed	3 (4.8)	4 (4.7)	
Region of contamination			<0.0001‡
Highlands region			
Analamanga	38 (60.3)	18 (21.2)	
Amoron’i Mania	8 (12.7)	4 (4.7)	
Bongolava	3 (4.8)	0 (0)	
Itasy	6 (9.5)	3 (3.5)	
Vakinankaratra	7 (11.1)	3 (3.5)	
Other	1 (1.6)¶	57 (67.1)	

*Mean (±SD) ages: sporotrichosis patients 38.1 (±19.5); other patients, 43.2 (±18.1); p = 0.10 (Student t test p value to compare mean age between both groups). †χ2 test for trend. ‡χ2 or Fisher exact test to compare categorical variables between both groups.  §On trunk, leg and arm, or leg and thorax. ¶Localized to Atsinanana.

The male sex predominance was less marked among patients with sporotrichosis (n = 44, 69.8%) than among the other patients recruited. This difference, however, was not significant (p = 0.29) (Table 2).

Patients with sporotrichosis were younger than other patients (38 vs. 43 years of age), although this difference was not statistically significant (p = 0.10). Analysis by age group showed that this trend was linked to a high frequency of sporotrichosis in persons <18 years of age (64.7%) compared with the rest of the population (39.7%; p = 0.08).

**Figure 2:**
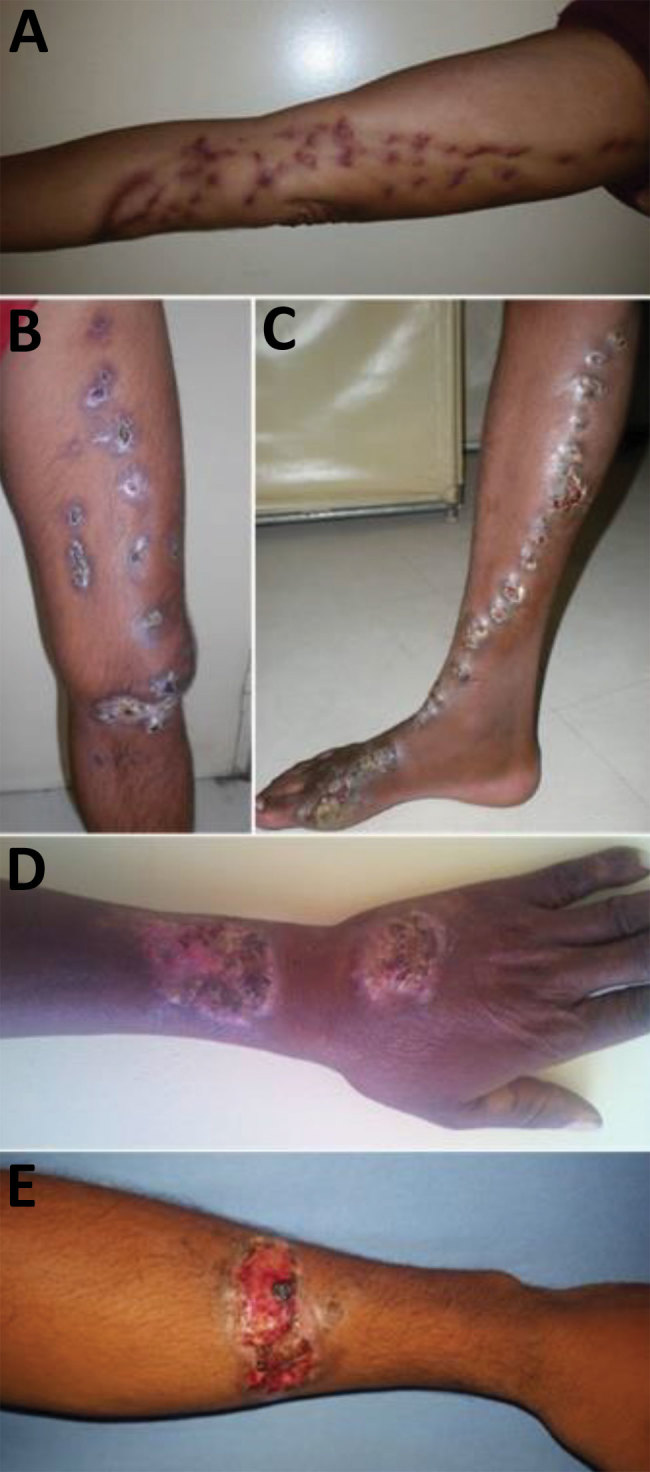
Clinical manifestations of sporotrichosis in patients with chronic cutaneous and subcutaneous lesions, Madagascar, March 2013–June 2017. A–C) Lymphocutaneous lesions. D) Lymphocutaneous ulcerative budding and crusty lesion. E) Ulceroerosive and erythematosus lesion with irregular border, easily misdiagnosed as chromoblastomycosis.

The location of lesions differed significantly between sporotrichosis patients and other patients (Table 2). The principal site affected was the arms (49.2%) for patients with sporotrichosis. Sporotrichosis was diagnosed more frequently for patients with lesions on the arm (73.8%) than for patients with lesions on the leg (32.3%) or other body sites (15.4%) (p<0.0001; Table 2). The sporotrichosis lesions had been present for <1 year for 71% of patients, 1–2 years for 14.5% of patients, and >2 years for 14.5% of patients. The most frequent type of lesion for sporotrichosis patients was lymphocutaneous (69.3%). The other forms were characterized by vegetative or verrucous lesions, infiltrated plaques, or tuberous lesions (Figure 2). We observed no fixed cutaneous forms or extracutaneous forms.

Sporotrichosis patients were predominantly farmers (52.4%), but a large number were craftsmen and tradesmen (Table 2). Sporotrichosis was diagnosed for 71.4% of the craftsmen and tradesmen and 39% of patients in other professions grouped together (p = 0.04).

### Prevalence and Geographic Distribution of Sporotrichosis

We determined the geographic origin of patients with sporotrichosis, corresponding to the presumed origin of contamination (Figure 1, panel C). The concentration of sporotrichosis cases in the highlands was very high; almost all patients with sporotrichosis (n = 62, 98.4%) originated from these areas. The frequency of sporotrichosis cases was much higher in the highlands (62/90, 68.9%) than on the rest of the island (1/58, 1.7%; p<0.0001). In all highland areas, the frequency of sporotrichosis was similar and very high (66.6%–100%; p = 1.00) (Table 2).

The average annual prevalence of sporotrichosis on the high plateaus of the Analamanga, Amoron’i Mania, Bongolava, Itasy, and Vakinankaratra regions was evaluated at 0.21 cases/100,000 inhabitants. Prevalence was highest in the Analamanga (0.27/100,000 inhabitants) and Amoron’i Mania (0.25/100,000 inhabitants) regions (Table 3; Figure 1, panel C).

**Table 3:** Prevalence of sporotrichosis in Madagascar, March 2013–June 2017

Region	Mean no. inhabitants/y*	No. cases, 2013–2017	Mean no. cases/y	Annual prevalence/100,000 inhabitants (95% CI)
North and North-Central: Analanjirofo, Sava, Sofia	3,443,999	0	0	0
Highlands	7,448,855	62	15.5	0.21 (0.2097–0.2103)
Analamanga	3,534,578	38	9.5	0.27 (0.2695–0.2705)
Amoron’i Mania	754,695	8	2.0	0.25 (0.2490–0.2510)
Bongolava	482,742	3	0.8	0.16 (0.1590–0.1610)
Itasy	773,490	6	1.5	0.17 (0.1692–0.1708)
Vakinankaratra	1,903,350	7	1.8	0.09 (0.0896–0.0904)
West: Melaky, Menabe, Boeny	1,774,661	0	0	0
East and Southeast	3,527,693	1	1.3	0.04 (0.0398–0.0402)
Alaotra Mangoro	1,084,092	0	0	0
Atsinanana	948,560	1	1.3	0.13 (0.1293–0.1307)
Vatovavy Fitovinany	1,495,041	0	0	0
South and Southwest: Androy, Anosy, Atsimo Andrefana, Ihorombe	3,203,165	0	0	0

*Mean over the period was calculated from the last figures available in 2013 adjusted for the subsequent years with a growth of 2.7% per year (World Bank estimates of the demographic growth in Madagascar, https://donnees.banquemondiale.org/indicateur/SP.POP.GROW).

### Mycologic Results

We collected 192 samples from the 148 patients. Direct examination yielded negative results for all cases, and we were unable to perform histologic examinations (Appendix 1).

### Culture Results

We obtained 172 cultures, including 72 established with the samples of the 63 sporotrichosis patients. Overall, macroscopic and microscopic examination exhibited morphologic features consistent with *Sporothrix* spp. for 90.2% (65/72) of the cultures.

### Molecular Results

Sensitivities for the 2 panfungal PCRs, for D1D2 and ITS, were lower for clinical specimens than for cultures, and the ITS PCR was less sensitive than the D1D2 PCR for clinical specimens and cultures (Appendix 2 Figure 1). The specific *S. schenckii* topoisomerase II PCR was unable to confirm identification for any of the clinical specimens, whereas its sensitivity for cultures was 89.2% (58/65), with features suggestive of *Sporothrix* spp.

**Figure 3:**
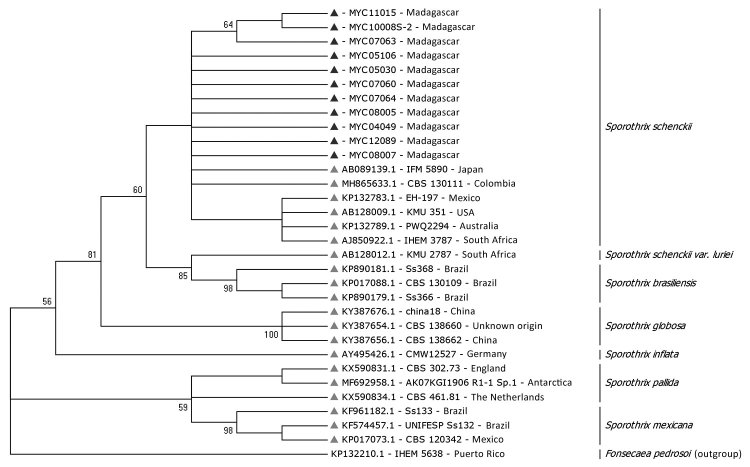
Phylogenetic tree of internal transcribed spacer sequences of *Sporothrix schenckii* isolates from patients with sporotrichosis, Madagascar, March 2013–June 2017 (black triangles), and reference isolates (gray triangles). *Fonsecaea pedrosoi* was considered to be out of group. The tree was built by using MEGA7.0 software (https://www.megasoftware.net) and applying the maximum-likelihood method based on the Kimura 2-parameter model (100 bootstrap replicates). Strains are detailed in Appendix 1 Table 1. GenBank accession numbers for isolates from this study: MYC11015, MK342563; MYC12089, MK342536; MYC10008-S2, MK342530; MYC08007, MK342562; MYC08005, MK342529; MYC07064, MK342535; MYC07063, MK342534; MYC07060, MK342533; MYC05106, MK342564; MYC05030, MK342531; MYC04049, MK249820.

The alignment of the ITS sequences from cultures identified 13 isolates as *S. schenckii*, according to data from the ISHAM database (Appendix 1 Table 1) (*17*). The phylogenetic analysis showed that the isolates from patients with sporotrichosis in our study were grouped in the *S. schenckii* clade, together with clinical strains from other regions of the world (Figure 3; Appendix 2 Figure 2). Our results confirm that ITS sequencing is suitable for separating the cryptic species of clinical importance from the strictly environmental ones (*25*).

The MSP in-house *Sporothrix* library performed well for *S. schenckii* identification; the score for 87.9% (29/33) of the strains was >2 and for 4 strains was >1.8. Unfortunately, 2 strains were contaminated by *Candida* spp. and could not be identified. For sporotrichosis identification, in-house MSPs were systematically the first choice in the list of MSPs, confirming their superiority for identification at the species level over the 2 MSPs in the Bruker database and the 1 MSP in the NIH database. Comparison of the spectra obtained by using the MSP in-house *Sporothrix* library with those obtained from identification to the external MSI platform indicated that the most likely identification was *S. schenckii* (Appendix 1 Table 2). Percentages of similarities were consistent with accurate identification to the species level (>20%) for 94.1% (48/51) (Appendix 1 Table 1); only 3 strains were not formally identified.

Taking together all results of the molecular analyses, we identified 53 *S. schenckii* strains: 51 by MALDI-TOF mass spectrometry, of which 46 were identified also by the specific *S. schenckii* topoisomerase II PCR and 13 were identified also by ITS sequencing. The 2 contaminated isolates not identified by MALDI-TOF mass spectrometry were confirmed by the specific *S. schenckii* topoisomerase II PCR.

### Susceptibility of *Sporothrix schenckii* to Antifungal Drugs and Patient Outcomes

A total of 46 *S. schenckii* isolates were culturable after thawing for MIC determination. We determined MICs and their geometric means for the 5 antifungal drugs tested (Table 4). Overall, of the *S. schenckii* isolates, the terbinafine MIC was low (≤0.25 μg/mL) for 82.3% (38/46), the itraconazole MIC was ≤1 μg/mL for 74% (34/46), and the posaconazole MIC was ≤1 μg/mL for 56.5% (26/46). However, 65% of strains had MICs ≥4 μg/mL for isavuconazole.

**Table 4:** MICs of 5 antimicrobial drugs for 46 *Sporothrix schenckii* isolates in the mycelium phase, from patients in Madagascar, 2013–2017*

Drug	No. isolates with each MIC, μg/mL								MIC, μg/mL		
	<0.25	0.5	1	2	4	8	>16		GM	50%	90%
Posaconazole	10	11	13	6	1	5	0		0.78	1	4
Isavuconazole	1	0	7	8	13	17	0		3.35	4	8
Amphotericin B	14	10	9	8	3	0	2		0.7	0.5	2
Itraconazole	4	10	12	6	9	2	3		1.43	1	4
Terbinafine	38	3	2	1	2	0	0		0.14	≤0.25	0.5

*MIC 50% and 90% represent the minimal concentrations of drug that inhibit the isolates by that percentage. GM, geometric mean.

All 63 patients received treatment, but only 32 were monitored for >2 months after treatment began (23 with lymphocutaneous disease and 9 with minor forms of disease). Among those patients, the response was complete or major for 15 (47%) after 4–7 months of treatment and minor for 17 (53%) after prolonged (9 months) treatment. The remaining 31 patients received treatment for 2 months and then did not return for follow-up visits.

## Discussion

This study provides recent epidemiologic data for sporotrichosis in Madagascar. We detected numerous cases and substantial endemicity despite previous reports of only sporadic cases or small series (*16*,*26**).* We estimated an average annual prevalence in the highlands of 0.21 cases/100,000 inhabitants; 98% of the cases were concentrated in that area. Among sporotrichosis patients in Madagascar, we highlight the high infection risk for young persons (<18 years of age) and the particularly high frequency of lesions on the arms. On the basis of this study, we were able to develop and routinely implement molecular analyses in Madagascar, enabling positive identification of *S. schenckii* for all confirmed cases.

The high estimated prevalence (0.27 and 0.25/100,000 inhabitants) in 2 highland regions (Analamanga and Amoron’i Mania) reveals the high transmission rates in this part of the island. To date, the only published series of sporotrichosis cases in Madagascar have described disease-endemic foci in the highlands, particularly in the Analamanga region (*16*), but the concentration of cases in the highlands that we observed was unexpected and striking. This almost exclusive distribution may be the result of climate conditions in this region, which differ from those on the rest of the island. This region has a tropical climate, with a mean temperature of 19.5°C and substantial rainfall, which probably favors development of fungi on plants and in the soil. A phylogeographic study focusing strictly on *S. schenckii* showed that this species was present in temperate (United States), hot and humid (South Africa, Australia, Colombia, and Venezuela), hot and dry (Australia and Uruguay), cool and dry (Peru and South Africa), and cool and humid (Uruguay) zones (*27*)*.* Findings of that study therefore seem to go against the notion of a single climatic factor. The frequency of sporotrichosis in some areas of the island to the west and southwest are unknown because these areas were not investigated; thus, sporotrichosis may not be totally distributed in the highlands. In addition, some cases could have been missed because of our mode of study recruitment, the low incomes of people living in remote areas, and the limited development of the healthcare system. The concentration of sporotrichosis in the highlands is probably not the result of better access to healthcare facilities because patients with other diagnoses most frequently do not live in the highlands (p<0.0001) (Table 2). However, better access to medical care in the highlands does partly explain the sporotrichosis diagnoses made relatively early in the course of disease (71% in the first year after onset).

Patients found it difficult to remember when their lesions had appeared and to associate them with a particular injury or activity; however, our survey revealed considerable involvement in rural activities: farming (rice, cassava, corn), logging, trade, and craftsmanship. Not only artisans are exposed to contamination through manual work; tradespeople are also exposed because they practice activities other than selling for living. Contamination by activities associated with the manual production of charcoal and cutting wood for cooking and heating seems likely on the basis of the predominance of arm lesions, the concentration of the disease in the coldest region of the island, and the high frequency of infections among children (who practice these activities). In northeastern China, a risk associated with the use of wood or other fuel has been proposed as an explanation for transmission patterns (*28*); the authors of that study thought that contamination occurred via maize stalks (where *S. globosa* has been found) used for heating and cooking. They hypothesized that the fermentation of the plants promotes the development of yeast forms of the fungus, increasing the risk for contamination during transport and storage at home.

Other possible sources of contamination, such as soil or decaying plant material, are also possible in Madagascar. Neither we nor J.F. Carod et al. (*16*) observed any cases of zoonotic transmission; the identification of *S. schenckii* alone confirms the hypothesis of contamination by soil and plants.

The molecular methods that we developed in this study, including MALDI-TOF mass spectrometry, made it possible to confirm cases and to identify the species responsible (*21*,*29*). We found that it was easier to amplify the D1D2 domain (LSU) and that the amplicons obtained were easier to analyze by sequencing than were those of the ITS domain. However, the availability of a database with many verified ITS sequences and the more polymorphic and discriminant nature of these sequences makes them more suitable for cryptic species identification and phylogenetic analysis (*17*,*25*).

We added a rapid and inexpensive mass spectrometry identification approach to the molecular tools for identifying *S. schenckii* to the cryptic species level. Our results obtained by using a Bruker instrument confirm previous analyses performed with a Shimadzu instrument (*29*). The MSP in-house *Sporothrix* library yielded better *S. schenckii* identification scores than did the 3 preexisting MSPs. The excellent identification scores and the external validation with another mass spectrometry platform showed that *S. schenckii* identification at the species level with MALDI-TOF mass spectrometry is accurate and adapted for routine diagnoses in clinical laboratories.

In conclusion, our study reveals substantial endemicity of sporotrichosis in Madagascar. Sporotrichosis was particularly concentrated in the highlands, which have climate, vegetation, and lifestyle conditions that favor the development and transmission of the fungus. Using molecular methods and MALDI-TOF mass spectrometry, we were able to identify *S. schenckii* as the species responsible for sporotrichosis in Madagascar. Despite its high frequency, sporotrichosis remains neglected in Madagascar.

Appendix 1 Details of identification of clinical isolates from patients with suspected sporotrichosis in Madagascar, 2013–2017.

Appendix 2 Results of panfungal PCR and phylogenetic tree of internal transcribed spacer sequences of *Sporothrix schenckii* isolates from patients in Madagascar, 2013–2017.
