# Supplementary material for: Sporotrichosis in the Highlands of Madagascar, 2013–2017
Source: Emerg Infect Dis. 2019 Oct;25(10):1893–902. doi: 10.3201/eid2510.190700 (PMC6759251; doi:10.3201/eid2510.190700)
Supplement: Appendix 2 — Results of panfungal PCR and phylogenetic tree of internal transcribed spacer sequences of Sporothrix schenckii isolates from patients in Madagascar, 2013–2017. [file 19-0700-Techapp-s2.pdf]

Article DOI: https://doi.org/10.3201/eid2509.190700

Sporotrichosis in the Highlands of Madagascar, 2013–2017

Appendix

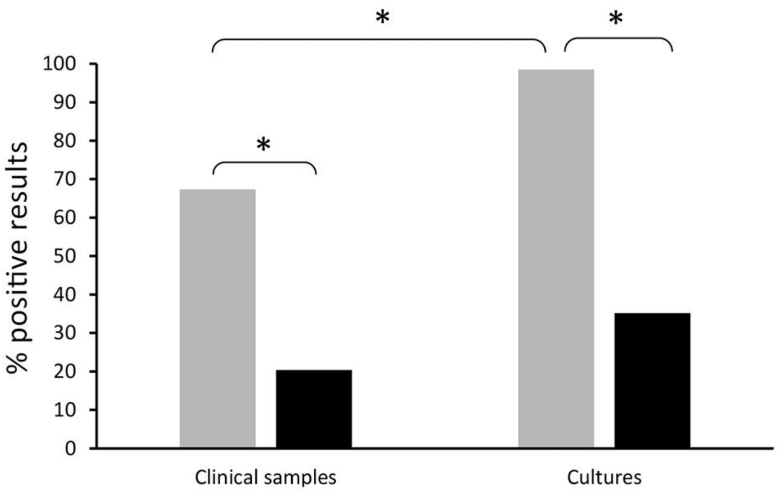

Appendix Figure 1. Results of panfungal PCR. The percentage of positive results for D1D2 (in gray) or ITS amplification (in black) is shown in the *y-axis*. *p<0.0001 in chi-squared tests

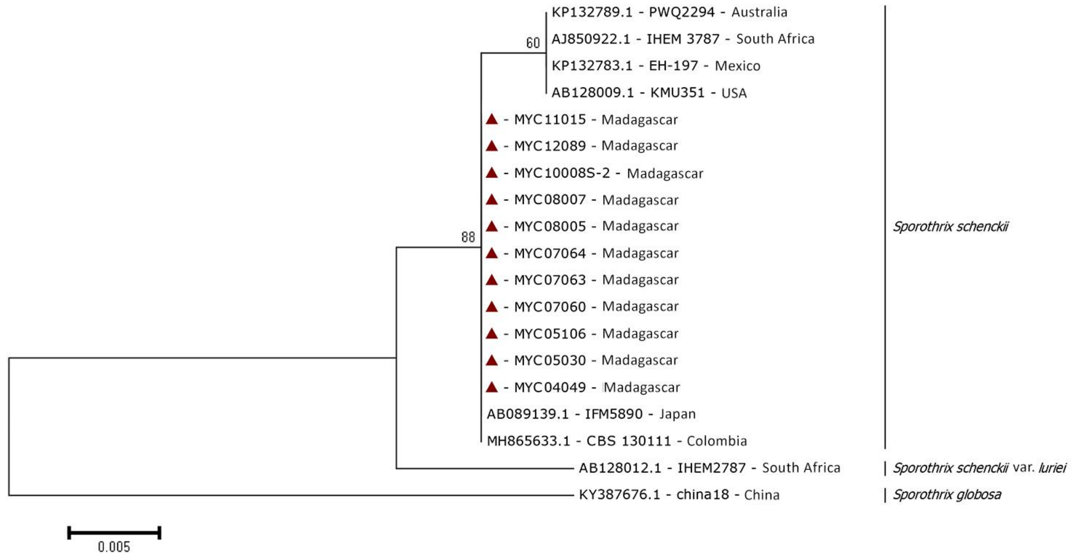

Appendix Figure 2. Phylogenetic tree of ITS sequences of *Sporothrix schenckii* isolates from patients in Madagascar. *S. globosa* was considered to be out of group. The tree was built using MEGA7.0 software

by applying the maximum likelihood method based on the “Tamura 3-parameter model” (100 Bootstrap replicates). Strains are detailed in Appendix 1 Table 1 (https://wwwnc.cdc.gov/EID/article/25/10/19-0700-App1.xlsx). ▲ Clinical strains used in this study, GenBank accession number: MYC11015: MK342563; MYC12089: MK342536; MYC10008-S2: MK342530; MYC08007: MK342562; MYC08005: MK342529; MYC07064: MK342535; MYC07063: MK342534; MYC07060: MK342533; MYC05106: MK342564; MYC05030: MK342531; MYC04049: MK249820.
